# I Want to Help You, But I Am Not Sure Why: Gaze-Cuing Induces Altruistic Giving

**DOI:** 10.1037/a0033677

**Published:** 2013-08-12

**Authors:** Robert D. Rogers, Andrew P. Bayliss, Anna Szepietowska, Laura Dale, Lydia Reeder, Gloria Pizzamiglio, Karolina Czarna, Judi Wakeley, Phillip J. Cowen, Steven P. Tipper

**Affiliations:** 1 School of Psychology, Bangor University, Bangor, North Wales, United Kingdom; 2 School of Psychology, University of East Anglia, Norwich, England; 3 Department of Psychology, University of Bath, Bath, England; 4 Faculty of Psychology, University of Warsaw, Warsaw, Poland; 5 Department of Psychiatry, University of Oxford, Oxford, England; 6 Department of Psychology, University of York, York, England

**Keywords:** gaze, joint attention, altruism

## Abstract

Detecting subtle indicators of trustworthiness is highly adaptive for moving effectively amongst social partners. One powerful signal is gaze direction, which individuals can use to inform (or deceive) by looking toward (or away from) important objects or events in the environment. Here, across 5 experiments, we investigate whether implicit learning about gaze cues can influence subsequent economic transactions; we also examine some of the underlying mechanisms. In the 1st experiment, we demonstrate that people invest more money with individuals whose gaze information has previously been helpful, possibly reflecting enhanced trust appraisals. However, in 2 further experiments, we show that other mechanisms driving this behavior include obligations to fairness or (painful) altruism, since people also make more generous offers and allocations of money to individuals with reliable gaze cues in adapted 1-shot ultimatum games and 1-shot dictator games. In 2 final experiments, we show that the introduction of perceptual noise while following gaze can disrupt these effects, but only when the social partners are unfamiliar. Nonconscious detection of reliable gaze cues can prompt altruism toward others, probably reflecting the interplay of systems that encode identity and control gaze-evoked attention, integrating the reinforcement value of gaze cues.

Social interactions frequently result in the formation and adjustment of opinions about the character of other people (Heider, 1958; E. E. Jones & Davis, 1965). Prominent models posit that these opinions center around two dimensions: warmth/emotion and competence/dominance (Todorov, Baron, & Oosterhof, 2008). Appraisals of the first of these dimensions can be expressed in judgments about the trustworthiness of individuals encountered in our social environment and can reflect emotional or structural cues in the face (Oosterhof & Todorov, 2009; Stirrat & Perrett, 2010). Some of the behavioral cues that underpin characterological judgments of this kind are overt and highly salient, such as continuous angry outbursts. However, other cues are subtle and require the integration of information over time, such as reevaluating character in the light of behavior that reveals deception or dishonesty. In many situations, such judgments are based upon little more than impressions gained in fleeting encounters in noisy social environments. Therefore, an important objective for social psychologists is to understand how people form impressions about others using subtle cues that may be encoded nonconsciously.

When interacting with another person, one salient source of information is the direction of his or her gaze. That is, there is an automatic simulation of the gaze direction of social partners. For example, if a person looks to the left, an observer’s attention automatically follows toward the left (i.e., toward the gazed-at location or object; Friesen & Kingtone, 1998; Frischen, Bayliss, & Tipper, 2007). This state of shared, or joint, attention facilitates processing of important objects in the environment; it is also fast and automatic, being difficult to override (Driver et al., 1999). This means that, as well as being an important source of information used to predict the future actions of other people via their current focus of attention, direction of gaze can be used to deceive (Emery, 2000). Illustrating the latter, directed gaze toward or away from favored environmental locations has been shown to influence competitive interactions between conspecifics searching for valuable food resources (Bugnyar & Heinrich, 2006; Bugnyar & Kotrschal, 2004). More prosaically, directing gaze (in order to deceive) can be observed in skillful basketball players or football players—such as Ronaldhino—who quickly look toward one teammate but simultaneously passes the ball in the opposite direction (see the video at http://www.youtube.com/watch?v=RulvevIFIpk).

Here, we describe five experiments that adapted the standard gaze-cuing procedure to investigate what is learned about social partners when we follow their direction of gaze (Bayliss, Frischen, Fenske, & Tipper, 2007; Bayliss & Tipper, 2006). Consider Figure 1A: Some individuals *always* look toward the location of objects presented soon afterward (“valid” faces). Response times (RTs) for categorizing targets presented in these locations are speeded, since these individuals’ direction of gaze provides reliably helpful information about the spatial location of the to-be-presented targets. By contrast, other individuals *always* “deceive” by looking away from the future location of target objects (“invalid” faces), slowing response times. Participants do not appear to be aware of these consistent person identity-gaze contingencies, and the magnitudes of these gaze-cuing effects are comparable to when individual faces look equally often toward and away from targets (Bayliss, Griffiths, & Tipper, 2009; Bayliss & Tipper, 2006).[Fig-anchor fig1]

There are three critical issues. First, does implicit acquisition of such gaze-cuing contingencies influence subsequent exchanges with people in economic games? Previous experiments indicate that reliable gaze-cuing can enhance explicit judgments about trustworthiness as measured by forced-choice procedures (Bayliss et al., 2009; Bayliss & Tipper, 2006). However, it is unknown whether reliable (or unreliable) gaze cues in other people can influence actual behavior in subsequent economic exchanges.

Second, what are the mechanisms that mediate these effects? One possibility is that reliable gaze cues enhance the probability estimates that particular individuals will respond generously in economic transactions (Behrens, Hunt, Woolrich, & Rushworth, 2008; R. M. Jones et al., 2011), raising the possibility that such individuals can be exploited in social exchanges. On the other hand, gaze contingencies might also evoke broader emotional reactions that could underlie generous, or affiliative, responses toward any individual whose gaze has been helpful or trustworthy in the past (Bayliss & Tipper, 2006). Possibly, such positive impulses generate obligations to behave fairly toward such social partners. Third, is learning or acquisition of the relationship between person identity and gaze contingency influenced by perceptual noise? The standard procedure for investigating gaze-cuing involves presenting a static head against uniform backgrounds and then generating gaze-shifts by lateral movements of the pupils/irises (e.g., Friesen & Kingstone, 1998). However, actual gaze detection typically takes place in noisy perceptual environments in which social partners are moving, sometimes unpredictably, and making rapid gaze shifts. Can we detect the effects of gaze cues upon economic exchanges between partners in such perceptually noisy conditions?

In the first experiment, we sought to establish whether helpful gaze cues enhance behavioral measures of trust in an adapted investment game (Fehr & Camerer, 2007). Participants who had previously completed the standard gaze-cuing task were asked to pass an amount of money to valid and invalid faces acting as trustees. The trustees invested the money, which was increased by some “market” process, before deciding how much money to pass back, as profit, to our participants. In this situation, assessments of trustworthiness are critical. We hypothesized that individuals who had previously looked toward targets (i.e., the valid faces) would be trusted to return more of the invested money than individuals who always looked away from targets (i.e., invalid faces), prompting higher value investments. That is, we sought to test whether patterns of eye-gaze that are implicitly encoded can alter subsequent behavioral (investment) manifestations of trust.

## Experiment 1: One-Shot Investment/Trust Games

### Method

Twenty healthy adult volunteers completed a standard gaze-cuing RT task (see below), followed by a series of amended one-shot investment/trust games and then, finally, explicit ratings of approachability and trustworthiness.

#### Participants

Ten males and 10 females participated. Their demographic and psychometric characteristics are set out in Table 1. All participants scored less than 9 on the Beck Depression Inventory (Beck, Ward, Medelson, Mock, & Erbaugh, 1961), indicating an absence of recent depressive symptoms (Beck, Steer, & Brown, 1996). Participants’ scores on the Autism Questionnaire (AQ) were also comparable with those reported in both community and student samples (Baron-Cohen, Wheelwright, Skinner, Martin, & Clubley, 2001). Finally, participants’ verbal IQs, estimated using the Mill Hill Vocabulary Scale (Raven, Court, & Raven, 1998), fell within the normal to superior range.[Table-anchor tbl1]

#### Standard gaze-cuing task

##### Stimuli

All stimuli were comparable to those used previously (Bayliss et al., 2009; Bayliss & Tipper, 2006). Sixteen faces from the NimStim face database (http://www.macbrain.org/resources.htm) were arranged in pairs matched for gender, ethnicity and approximate age. The face stimuli comprised two pairs each of Black males and Black females and three pairs each of White males and White females. In each experiment, pairs of faces were allocated to Face Group A or Face Group B. Previously, 12 independent raters ensured that all pairs of faces were rated for equal attractiveness and trustworthiness; and that, as a whole, both sets of faces (A and B) were approximately equal in age, attractiveness and trustworthiness (Bayliss et al., 2009).

Three versions of each face were produced, one with a direction of gaze straight ahead so that the face looked directly at participants when presented in the middle of the computer display, one with the pupils averted leftward, and another with the pupils averted rightward. The faces measured approximately 10.6 cm × 10.0 cm. Eye regions measured between 4.0 cm and 4.5 cm from the left corner of the left eye and the right corner of the right eye. The eyes measured approximately 0.5 cm × 1.0 cm, with pupils/irises of approximately 0.5 cm × 0.5 cm. As previously (Bayliss, Schuch, & Tipper, 2010), all faces held a moderate smiling expression and were initially presented looking straight ahead. Manipulations of the faces allowed the eyes to appear to look toward the left or look toward the right.

The target stimuli comprised pictures of 36 household objects. Eighteen objects were categorized as belonging in the kitchen, and 18 objects were categorized as belonging in the garage. The objects appeared in red, blue, green, or yellow and in two orientations (e.g., handles of objects on the left or right), yielding 288 stimuli. Targets varied between 1.5 × 3.0 cm and 5.0 × 8.0 cm and were presented centered at 10.0 cm toward the left or toward the right of the center of the screen (Bayliss, Paul, Cannon, & Tipper, 2006).

##### Procedure

**Figure 1 fig1:**
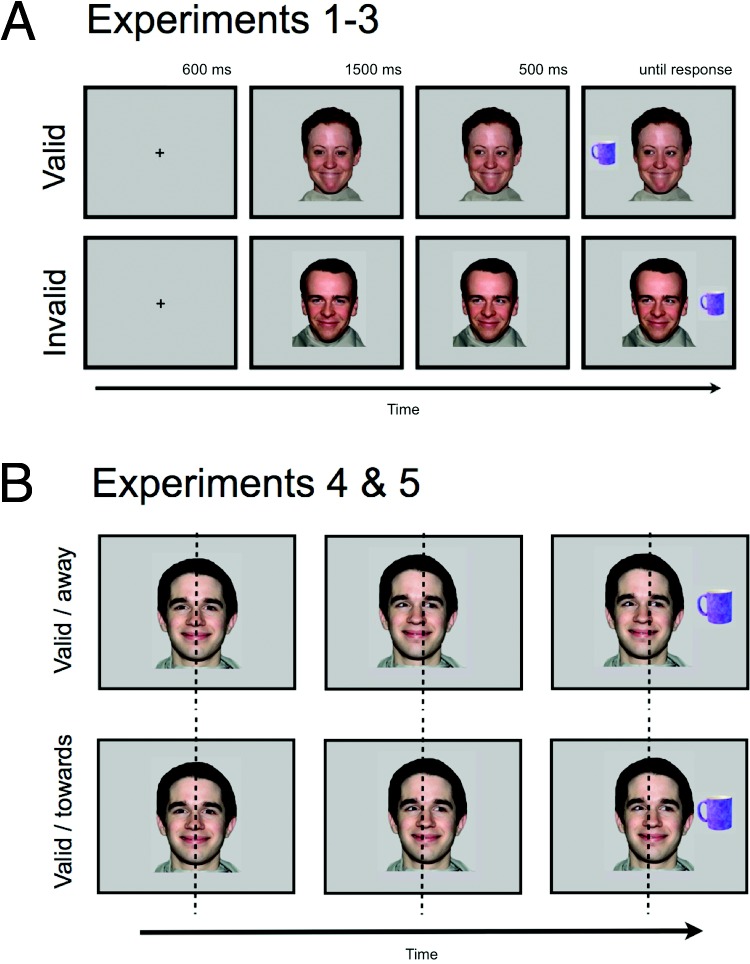
A: Trial structure for the standard gaze-cuing task in which the eyes shifted toward the left or right side of the displays (used in Experiments 1, 2, and 3). B: Trial structure for the amended gaze-cuing task in which the head, shifted randomly toward the left or right side of the display (Experiments 4 and 5). Head translation toward and away from the target location are shown for a single, “valid” face. Dashed line indicates midline for illustration only and was not present on the screen. Head translation was approximately 0.8 cm and is not shown here to scale. Valid faces were followed by objects presented on the same side as the gaze-shift; invalid faces were followed by objects on the opposite side. The images were taken from the NimStim face database (http://www.macbrain.org/resources.htm).

Participants were seated, centrally and at an appropriate height, in front of a standard computer display. Task stimuli were presented at a distance of approximately 60 cm. At the start of each trial, participants fixated a central cross while covering two response keys with the forefinger and thumb of their dominant hand. Following a delay of 600 ms, the cross was replaced by a face (see Figure 1A). After another 1,500 ms, the eyes of the face looked toward the right or left. A household object appeared 500 ms later on the left or right of the display. Participants were instructed to decide, as quickly and accurately as possible, whether the object belonged (typically) in the garage (“h” key) or kitchen (spacebar key). Auditory feedback followed: bell = correct; buzzer = incorrect. If no response was made after 2,500 ms, the trial was coded as an error, and the next trial presented. Finally, a blank screen was displayed for a 1,500-ms intertrial interval (ITI).

Participants completed 192 trials (two blocks of 96 trials), with eight “valid” (eyes moving toward the target objects) and eight “invalid” (eyes moving away from the targets) faces appearing 12 times in a randomized order, paired with randomly selected targets. For half of the participant sample, the eight valid faces were taken from Face Group A and the eight invalid faces were taken from Face Group B; for the other half of sample, this assignment was reversed. Participants completed 16 practice trials with a single novel face.

##### Data analysis

Mean correct RTs and error proportions from the gaze-cuing task were analyzed using repeated-measures ANOVAs with the two between-subjects factors of participant-gender and Face Group (A vs. B) and the single within-subject factor of cue (valid vs. invalid faces). All trials with RTs longer than 1,500 ms were excluded from the data analyses. Error proportions were arcsine-transformed, as is appropriate whenever the variance of a measure is proportional to its mean (Howell, 1987). However, the error rates are reported as untransformed percentages in the text.

#### One-shot investment/trust games

These games were adapted from those used previously (King-Casas et al., 2005; Kosfeld, Heinrichs, Zak, Fischbacher, & Fehr, 2005). Participants were invited to make single investments of between £1 and £10 with each of the 16 faces seen in the earlier gaze-cuing task. Participants were told that the individuals shown in the photographs had previously attended the laboratory for a different experiment but had agreed to participate as trustees in the one-shot investment/trust games of the current experiment. This involved the experimenters contacting the trustee to indicate the profit earned by the participant’s investment on one randomly chosen game and then allowing the trustee to choose how much of the profit to return to the participant as the investor.

In each game, a valid or invalid face, with gaze straight ahead, was displayed in a central position above the caption “Here is Trustee *n*,” where *n* equaled 1–16 faces. Two seconds later, participants were prompted to press the spacebar to continue, ensuring that they had attended to (and properly processed) the presented face. The caption was then re-presented to read “You have £10. How many pounds do you want to invest with this trustee?” (see Figure S1 in the online supplemental materials). Participants entered an amount between £1 and £10 (in whole £s only) using a standard keyboard. Following this, the caption was re-presented as “You have invested £a; You have kept £10–*a*” (where *a* refers to the amount invested). In the full version of the investment/trust game, the caption was re-presented again, 3 s later, to state “Trustee *n*. This investment is now worth £*b*,” with *b* being a sum incremented every 300 ms to eventually reach three times the monetary value of the original investment. At this point, the final caption was presented as “Trustee *n*; You have invested £*a*; This trustee will repay £*c*; This trustee will keep £*b–c*,” where *b-c* was shown an incrementing number to indicate a large (generous) or a small (mean) return. This display remained for 3 s, after which participants pressed the spacebar for a blank ITI of 3 s.

Participants played four practice games with novel faces using the full version of the game. However, when making investments with the eight valid and the eight invalid faces of the gaze-cuing procedure, the final stage of the game in which the investments increased before trustees provided returns to participants, was omitted. This ensured that participants’ investments were not influenced by variability in different trustees’ payoffs.

Pilot testing had demonstrated that the range of investments offered to participants when deciding how much to invest with each face (i.e., between £1 and £10) elicited sufficiently variable behavior to facilitate the detection of different patterns of investment to the valid and invalid faces. Participants were also informed that, at the end of the experiment, one investment made with a valid or an invalid faces would be chosen at random and paid out for real; the return being added to participants’ attendance fees.

##### Data analysis

Previous evidence suggests gender-specific differences while playing economic games (Eckel & Grossman, 1998). Therefore, mean investments in the one-shot investment/trust games were tested by analysis of variance (ANOVA) with the two between-subjects factors of participant-gender and Face Group, and the single within-subject factor of cue (valid vs. invalid faces). The proportion of participants making larger or smaller investments with valid compared to invalid faces was assessed with a standard binomial test. Associations between differences in investments made to valid and invalid faces, on the one hand, and gaze-cuing effects (expressed as mean RTs for object categorizations following invalid faces—mean RTs for categorizations following valid faces) and AQ scores, on the other hand, were tested with Pearson’s coefficients.

#### Trustworthiness and approachability ratings

Finally, following completion of the gaze-cuing procedure and the one-shot investment/trust games, participants were again shown the eight valid and eight invalid faces presented in the gaze-cuing task, this time with their gaze straight ahead. The caption “How much do you trust this player? Please select a number on the keypad” was displayed at the bottom of the display for 3,000 ms. Participants provided ratings using a Likert-type scale of 1 to 7, using the computer keyboard. Blank screens were presented for ITIs of 3 s. Participants repeated this procedure with the same 16 faces, and the caption of “How readily would you approach this player? Please select a number on the keypad.”

##### Data analysis

Mean trustworthiness and approachability ratings were analyzed using repeated-measures ANOVAs with the between-subjects factors of participant-gender and face group, and the single within-subject factor of cue (valid faces vs. invalid faces).

#### Debriefing

All participants were questioned in order to identify and exclude participants who perceived correctly that particular (i.e., valid) faces in the gaze-cuing task shifted their gaze consistently toward the same side of the display as the to-be-categorized objects while other (i.e., invalid) faces shifted their gaze to the opposite side of the display.

### Results

**Figure 2 fig2:**
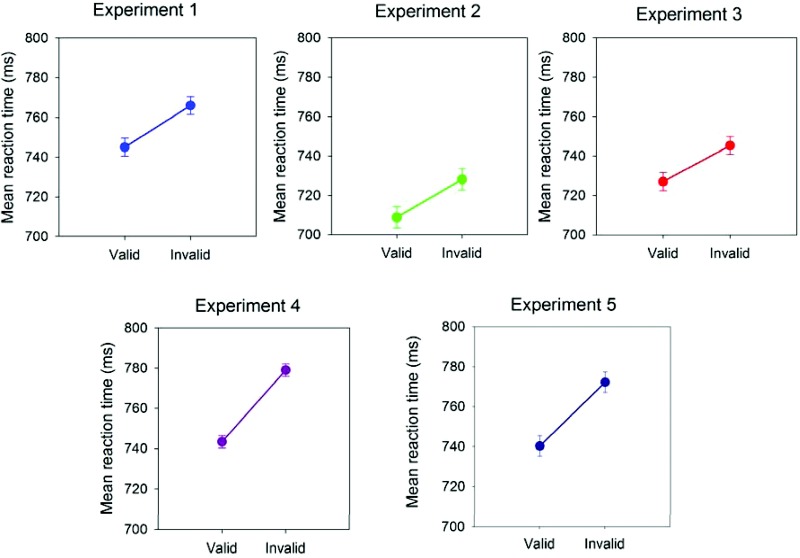
Mean correct reaction time (in milliseconds) for target categorizations (“kitchen” vs. “garage” objects) presented following valid and invalid faces in the gaze-cuing paradigm in Experiments 1, 2, 3, 4, and 5. Error bars represent standard errors of the means calculated for within-subject designs (Loftus & Masson, 1994).

Participants’ RTs while categorizing kitchen and garage objects were significantly faster following presentation of the valid faces compared to the invalid faces (see Figure 2), *F*(1, 18) = 10.70, *p* < .004, partial η^2^ = .37. There was no marked or significant change in error rates following valid compared to invalid faces (2.65 ± 0.54% vs. 2.60 ± 0.55%; *F* < 1), partial η^2^ < .01.[Fig-anchor fig2]

**Figure 3 fig3:**
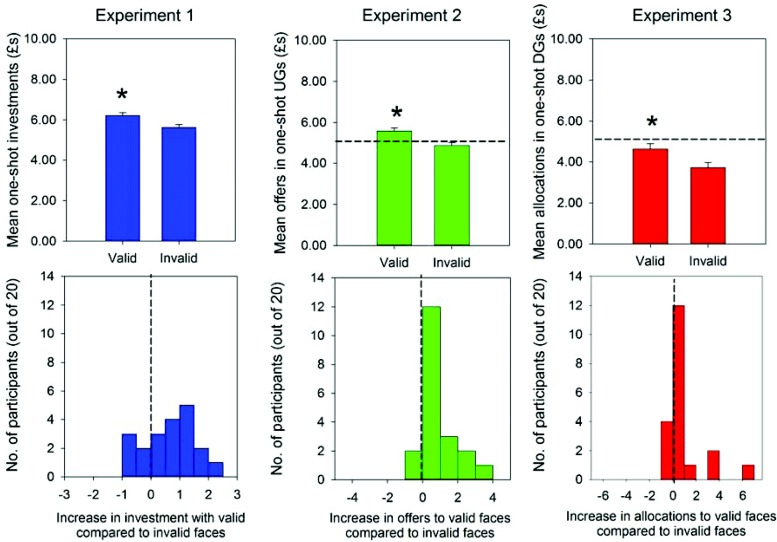
Top row: mean investments with valid and invalid faces in the one-shot investment/trust games of Experiment 1, mean offers to the valid and invalid faces of the one-shot ultimatum games of Experiment 2, and mean allocations to the valid and invalid faces of the one-shot dictator games of Experiment 3. Error bars represent standard errors of the means calculated for within-subject designs (Loftus & Masson, 1994); horizontal dashed lines represent equal offers to valid and invalid faces in one-shot ultimatum and dictator games of Experiments 2 and 3. Bottom row: frequency of investment increases (and decreases) to valid compared to invalid faces across participant sample in Experiment 1, frequency of offer increases (and decreases) across participant sample of Experiment 2, and frequency of allocation increases (and decreases) across participant sample of Experiment 3. Vertical dashed lines represent equal frequency of investment increases (or decreases) to valid over invalid faces in participant sample of Experiment 1, equal frequency of offer increases (or decreases) to valid over invalid faces in participant sample of Experiment 2, and equal frequency of allocation increases (or decreases) to valid over invalid faces in participant sample of Experiment 3. * *p* < .05.

Debriefing confirmed that none of the participants in Experiment 1 were aware that some faces of the gaze-cuing task always looked toward the subsequent targets whilst others always looked away from the targets, suggesting that investments made with valid and invalid faces reflected implicit processes. Participants invested significantly more money with the valid compared to the invalid faces (Figure 3, top row), *F*(1, 16) = 8.13, *p* = .012, partial η^2^ = .34. Inspection of individual participants’ behavior showed that 15 out of 20 made larger investments to the former relative to latter faces (Figure 3, bottom row; *p* = .041).[Fig-anchor fig3]

The enhanced investments made to valid compared to invalid faces were not associated with gaze-cuing effects (*r* = –.01); neither were they associated with participants’ AQ scores (*r* = .10). Investments were not substantially influenced by gender (*F* < 1), η = .06, or by the specific set of faces assigned as valid or invalid, *F*(1, 16) = 1.21, partial η^2^ = .07. Finally, comparison of participants’ explicit ratings of the trustworthiness and approachability of valid and invalid faces revealed no significant differences (Table 2; *F*s < 1), partial η^2^s < .05.[Table-anchor tbl2]

### Discussion

Experiment 1 demonstrated, for the first time, that completion of the standard gaze-cuing procedure (Bayliss et al., 2009; Bayliss & Tipper, 2006) increased participants’ monetary investments in single encounters with valid faces when compared with invalid faces. This behavior reflected implicit processing since our participants were not aware that some faces, but not others, provided gaze cues that had previously helped cognitive performance; neither were they accompanied by changes in the explicit appraisal of trustworthiness and approachability. Therefore, the results of Experiment 1 indicate that the implicit detection of reliable gaze cues in other individuals can enhance behavioral manifestations of trust in subsequent economic transactions.

The next question is whether we can learn more about the mechanisms that mediate these effects. On the one hand, the one-shot investment/trust games of Experiment 1 involved a situation in which participants’ behavior could reflect exclusively instrumental reasoning about which individuals are, and are not, going to prove to be the most generous trustees in returning investment profits. On the other hand, it is possible that processing other people’s reliable gaze cues may generate other social, or affective, appraisals about them as social partners. In principal, these appraisals may be detectable in social exchanges that involve judgments about what is fair and what is unfair.

Experiment 2 tested this idea by examining how completion of the gaze-cuing procedure influenced subsequent behavior in adapted one-shot ultimatum games (UGs) with valid and invalid faces (Güth, Schmittberger, & Schwarze, 1982). In one-shot UGs, one individual (“the proposer”) is given £10 and asked to offer some proportion of this money to another individual (“the responder”). If the responder agrees to accept this offer, the money is paid out according to the proposer’s intentions; however, if the responder rejects this offer, neither party receives anything. Noncooperative models of behavior in UGs indicate that proposers should offer, and responders should accept, the minimal offer possible, realizing a Nash equilibrium (Camerer, 2003a). However, substantial cross-cultural evidence indicates that responders frequently reject, as unfair, any offers of less than 30%–40% of the money available; while proposers tend to offer responders significantly more than the minimum allowed (Camerer, 2003a; Oosterbeek, Sloof, & Van De Kuilen, 2004). Proposers’ generosity can reflect affective processes, motivations to fairness (Haselhuhn & Mellers, 2005; van’t Wout, Chang, & Sanfey, 2010) and even, perhaps, the simulation of respondents’ emotional reactions while estimating what might be considered to be fair or unfair offers made by the proposers (Frith & Frith, 2006).

In Experiment 2, participants completed the standard gaze-cuing procedure as above and then made proposals to split £10 between themselves and each of the valid and invalid faces in a series of single-shot UGs. If completing the gaze-cuing task induces merely instrumental judgments about the likelihood of good payoffs from individuals whose gaze has previously benefited cognitive performance, we might expect to see participants seeking to exploit the valid faces by making minimal or less generous offers compared to the offers made to the invalid faces. On the other hand, if completing the gaze-cuing task induces prosocial appraisals about what reliable or trustworthy individuals actually deserve, we might see the more generous offers to the valid faces.

## Experiment 2: (Proposing in) Ultimatum Games (UGs)

### Method

Twenty healthy adults (10 males; 10 females) completed the same gaze-cuing task as used in Experiment 1, followed by a series of one-shot ultimatum games (playing as proposers) and explicit ratings of approachability and trustworthiness. Participants were assessed and debriefed in the same way as Experiment 1 (Table 1).

#### One-shot ultimatum games (UGs)

These games were adapted from one used previously (Sanfey, Rilling, Aronson, Nystrom, & Cohen, 2003). Participants were invited to play one-shot UGs with each of the 16 photographed faces used in the gaze-cuing task, having been told that the individuals in the photographs had attended the laboratory for a different experiment but agreed to participate as respondents in the one-shot UGs. Participants were told that the experimenters would subsequently contact the responder in one randomly chosen UG and ask him or her to decide whether to accept or reject the participant’s offer.

In each UG, a valid or invalid face was presented in the center of the display above the caption “Here is person *n*”, where *n* equaled 1–16 faces. Two seconds later, participants pressed the spacebar to continue, helping to ensure that participants had paid attention to the presented face. Following this, participants responded to the question “You have £10. How much money would you like to offer this person?” (see Figure S1). Participants entered an amount between £1 and £10 (in whole £s only) using the number keys of the computer keyboard. To facilitate comparisons with the one-shot investment/trust games of Experiment 1, offers of less than £1 were unavailable. Once participants entered their offer, the caption underneath the valid or invalid face was re-presented as “You have given £*a*, you have kept £10 − *a*” where *a* indicated the amount offered; 3 s later, a blank screen was presented for an ITI of 3 *s.*

Participants were informed that, at the end of the experiment, a single one-shot UGs (with a valid or invalid face) would be chosen at random and paid out for real; the money participants elected to keep (rather than offer) being added to their attendance fees if the responders accepted their offer. Participants completed four practice games with faces not seen in the gaze-cuing task.

##### Data analysis

Mean offers in the one-shot UGs were tested by repeated-measures ANOVA with the between-subjects factors of participant-gender and Face Group, and the within-subject factor of cue (valid vs. invalid face). The numbers of participants making mean offers to the valid faces that were larger, smaller or equal to mean offers to the invalid faces (to two decimal places) was assessed with a standard χ^2^ test. Mean offers to the valid and invalid faces were also tested against a baseline of £1 and an even-split of £5 with one-sample tests. Associations between changed offers to the valid compared to invalid faces, gaze-cuing effects and AQ scores were tested with Pearson’s coefficients.

### Results

As above, participants were significantly faster to categorize the kitchen objects and garage objects following presentation of valid faces compared to invalid faces (see Figure 2), *F*(1, 39) = 6.51, *p* = .020, partial η^2^ = .29. There were no marked difference in errors following valid compared to invalid faces (3.05 ± 0.45% and 2.85 ± 0.39%; *F* < 1; partial η^2^ = .01).

Replicating Experiment 1, none of the participants were aware that some faces, but not others, reliably cued the location of the to-be-categorized targets. In the one-shot UGs, participants’ offers to both the valid faces and invalid faces were significantly greater than the minimal offer of £1, *t*(19) = 20.73, *p* < .0001 and *t*(19) = 18.83, *p* < .0001, respectively. However, participants’ offers to valid faces were still significantly more generous than their offers to invalid faces (Figure 3, top row), *F*(1, 16) = 10.71, *p* = .005, partial η^2^ = .40. Fourteen participants made larger offers to the valid faces compared to the invalid faces; while only two made smaller offers, and four made (overall) equal-sized offers (Figure 3, bottom row), χ^2^ = 12.40, *p* = .041. Offers to the valid, but not invalid faces, were also significantly greater than the even-split of £5, *t*(19) = 2.60, *p* = .017 and *t*(19) = 0.858.

As before, the increase in offers made to the valid compared to the invalid faces were not significantly associated with the magnitude of participants’ gaze-cuing effects (*r* = –.05), participants’ AQ scores (*r* = –.12), were not influenced by gender (*F* < 1, partial η^2^ = .02), or by which particular faces were assigned as valid or invalid (*F* < 1, partial η^2^ = .001). Finally, as in Experiment 1, participants’ explicit ratings of the trustworthiness and approachability of valid and invalid faces were not markedly different (see Table 2; *F*s < 1, partial η^2^s < .04).

### Discussion

Experiment 2 tested between two hypotheses about how completion of the gaze-cuing procedure would influence individuals’ offers in a series of one-shot UGs involving valid and invalid faces. The results showed that participants did not seek to exploit trusted individuals by making smaller offers to valid faces compared to the invalid faces. Rather, we found that participants made more generous offers to valid faces; offers that were reliably more generous than 50:50 splits of the £10 available. As in Experiment 1, this behavior was not associated with explicit awareness that the gaze of valid faces had facilitated performance in the categorization task; neither was it accompanied by changes in the explicit (or conscious) ratings of trustworthiness or approachability of valid faces.

Other evidence indicates that offers made by proposers while playing UGs can reflect emotional states (van’t Wout et al., 2010). The generous offers observed in the one-shot UGs of Experiment 2 suggest that our participants were disposed, or felt obligated, to make larger offers toward the valid faces than the invalid faces. The additional observation that these offers were significantly higher than an even split of £5 of the £10 suggests an affiliative impulse toward the valid faces. However, offers in the one-shot UGs could still reflect the self-interest associated with making offers that are large enough to be accepted by the responders but no larger than necessary (Camerer, 2003a). Therefore, in our third experiment of the series, we tested whether completion of the standard gaze-cuing procedure influenced monetary transactions in which this constraint is removed, and the behavior of the proposer is not dependent in any way upon the actions of the responders.

One such situation is the one-shot dictator game (DG; Bolton, Katok, & Zwick, 1998). In these games, individuals are given an endowment (e.g., £10) and simply invited to allocate some proportion of this money to another person. The money is split as proposed; the recipient has no ability whatsoever to influence the outcomes. Allocations in DGs are increased, or made more probable, in the presence of eye-like stimuli making eye “contact” with participants (Haley & Fessler, 2005; Nettle et al., 2013). However, it is unknown whether DG allocations are influenced by the reliability of gaze cues themselves.

In Experiment 3, participants completed the gaze-cuing task and then allocated £10 between themselves and the valid and invalid faces in a series of amended single-shot DGs. Generosity in the context of one-shot DGs is typically taken to indicate (painful) altruism (Camerer, 2003a). If completing the gaze-cuing task induces an obligation to altruism, even while there is no underlying instrumental (monetary) motivation, we should see more generous allocations to valid faces compared to invalid faces.

## Experiment 3: (Proposing in) Dictator Games (DGs)

### Method

Twenty healthy adults (10 males and 10 females) completed the same gaze-cuing task as used in Experiments 1 and 2, followed by a series of one-shot DGs and, finally, explicit ratings of approachability and trustworthiness. Participants completed the same assessments as in Experiments 1 and 2 (Table 1) and were debriefed in the same way.

#### One-shot dictator games (DGs)

This game was adapted from previous experiments (Camerer, 2003a). Participants were invited to play a series of one-shot DGs with each of the 16 faces shown as part of the gaze-cuing task. Participants were told that the experimenters would add the value of their allocation in one randomly chosen DG to their own and the recipient’s attendance fees.

In each game, a valid or invalid face was presented in the center of the computer display above the caption “Here is person *n*,” where *n* equaled 1 through 16 faces. Two seconds later, participants were prompted to press the spacebar to continue, ensuring that participants attended toward (and processed) the presented face. Following this, participants responded to the statement (Figure S1) “You have £10. How much money would you like to offer this person?” Participants entered an amount between £1 and £10 (whole £s only) using the computer keyboard. Following this, the caption was re-presented as “You have given £*a*, you have kept £10 − *a*,” where *a* indicated the amount of money allocated; 3 s later, a blank screen was presented for an ITI of 3 *s.* Participants completed four practice games with faces not seen in the gaze-cuing task.

##### Data analysis

Allocations in the one-shot DGs were tested by an ANOVA with the between-subjects factors of participant-gender and face group, and the within-subject factor of cue (valid vs. invalid). The numbers of participants making larger, smaller or equal offers to valid faces relative to invalid faces was assessed with a standard χ^2^ test. Mean allocations were tested using one-sample *t* tests against baseline of £1 and even-splits of £5. Associations between changed allocations to valid compared to invalid faces, gaze-cuing effects and AQ scores were tested using Pearson’s coefficients.

### Results

Again, and as in Experiments 1 and 2, participants’ were significantly quicker to categorize the kitchen and the garage objects following presentation of the valid faces compared to the invalid faces (see Figure 2), *F*(1, 18) = 7.97, *p* = .011, partial η^2^ = .30. Error rates were not substantively altered (4.40 ± 0.66% vs. 4.40 ± 0.95%; *F* < 1, partial η^2^ < .01).

None of the participants in Experiment 3 were aware that some faces always looked in the direction of the targets, whilst others always looked away from the targets. However, in the one-shot DGs, participants made significantly more generous allocations to the valid than invalid faces (Figure 3, top row), *F*(1, 16) = 6.34, *p* = .023, partial η^2^ = .28. Twelve participants made larger offers to the valid faces compared to invalid faces, four participants made smaller offers, and four made (overall) equal-sized offers (Figure 3, bottom row; χ^2^ = 12.40, *p* = .041). Participants’ allocations to all faces tended to be *less* than £5 or 50% of the money available, significantly so in the case of invalid faces, *t*(19) = −2.56, *p* = .019.

The increased allocations to valid over invalid faces were not associated with participants’ gaze-cuing effects (*r* = –.21) or AQ scores (*r* = .14). They were also not markedly different between the genders (*F* < 1, partial η^2^ = .01) or the faces assigned as valid or invalid (*F* < 1, η < .01). Finally, participants’ explicit ratings of the approachability and trustworthiness of the valid and invalid faces were not significantly different (Table 2), *F* < 1 and *F*(1, 16) = 1.13, partial η^2^s < .23.

### Discussion

Experiment 3 demonstrated that completion of the gaze-cuing procedure induced participants to make more generous allocations to valid compared to invalid faces in a series of one-shot DGs. As in Experiment 2, this behavior was apparent even though participants were unaware of the difference between the two sets of faces (in terms of one set of faces reliably cuing the spatial locations of subsequent to-be-categorized objects) and even though participants provided explicit judgments of the valid and invalid faces as equally trustworthy and approachable. In other words, in line with Experiments 1 and 2, the more generous allocations made to valid faces reflected implicit social processes.

Consistent with previous results (Camerer, 2003a), participants’ offers in the one-shot DGs of Experiment 3 were, in general, significantly larger than the minimal offer but markedly reduced compared to participants’ offers in the one-shot UGs of Experiment 2. In one-shot DGs, in contrast to ones-shot UGs, the allocation of money is not dependent in anyway upon the behavior of the recipients; the critical decisions are made by the participants operating as “dictators.” This means that the “dictators” can keep as much, or as little of, the money for themselves; their behavior is not constrained by the requirement to make allocations that are large enough to ensure that the respondents accept but not so large that they disadvantage the dictators unnecessarily. For these reasons, generous offers in DGs cannot reflect proximal monetary self-interest but could reflect the subjective value associated with altruism or the reputation for altruism (Zaki & Mitchell, 2011). Experiment 3 demonstrated that healthy individuals make more generous allocations to social partners who have previously shown reliable gaze cues, reflecting heightened altruism toward individuals who have been helpful previously or, at least, the need to maintain reputations for altruism when encountering reliable or previously helpful individuals.

Thus far, three separate experiments have confirmed that the reliability of people‘s gaze cues influence behavior in subsequent economic games. Clearly, during the standard gaze-cuing procedure, participants learn the relationships between individual faces and the way that they can prove to be helpful or unhelpful. However, the generality of these findings is limited by the static faces presented in the experiments so far. Social encounters typically occur in very dynamic environments, raising the possibility that the effects of gaze-cuing upon behavior in economic games might not replicate in perceptually noisy conditions. Furthermore, we already know that the neural mechanisms that underlie face-identity representation and gaze perception are dissociable (Hoffman & Haxby, 2000), with occipital and fusiform regions coding identity and recognition (Andrews & Ewbank, 2004) but parietal and superior temporal sulcal regions coding for gaze direction (Calder et al., 2007). The gaze-cuing effects demonstrated so far must involve linking face identities to the reinforcement value of valid and invalid faces. These complex associative processes, requiring as they do, the integration of information from two partly separate systems, may be vulnerable to perceptual disruption.

Therefore, we sought to identify a potential important boundary condition in the effects of gaze-cuing upon behavior in economic games. In Experiment 4, we introduced a small translation of the face stimulus toward the right or the left concurrently with the gaze cue. That is, the eyes might have looked left, but the head could have moved a small distance (i.e., a pupil’s width) to the left or to the right, independent of the gaze shift. In the context of our design, this meant that the *eyes* of the valid faces always looked toward the location of the to-be-categorized object (as in Experiments 1, 2, and 3), but the *face itself* may have moved slightly toward *or* away from that location in a nonpredictive manner. Similarly, while the eyes of an “invalid” face always looked away from where the to-be-categorized object would appear, the *face itself* was equally likely to have moved toward or away from that location. Thus, each face simultaneously provided two cues: one predictive and one nonpredictive. Previous work shows that these uncorrelated translations of the face do not interfere with the attentional effects of gaze-cuing (Bayliss, di Pellegrino & Tipper, 2005). Therefore, we predicted that the standard gaze-cuing effect upon object categorization would again be observed under these conditions. However, this form of perceptual noise may disrupt the effects of predictive gaze-cuing upon behavior in economic games, equalizing offers to valid and invalid faces in a subsequent series of one-shot DGs.

We also strengthened Experiment 4 in two further ways. First, standard implementations of one-shot DGs allow players to make allocations of zero (out of their endowments) as an expression of economically optimal behavior (Bolton et al., 1998; Camerer, 2003b). In order to facilitate comparisons with Experiments 1 and 2, the participants of Experiment 3 were obliged to allocate at least £1 (of the £10 endowment) to the valid and invalid faces in the earlier one-shot DGs. In Experiment 4, this restriction was dropped so that our participants were entirely free (as the dictators in the games) to give as much, or as little, as they wished to the valid faces and to the invalid faces as a stronger test of the capacity of gaze cues to influence altruistic behavior in economic transactions.

Second, Experiments 1, 2, and 3 demonstrated that participants favored the valid faces over the invalid faces in three different economic games even though they were unable to (self)-report any awareness of the underlying gaze-cue contingencies, suggesting the operation of implicit social processes. Nonetheless, participants might have been able to make accurate predictions about which faces reliably cued the location of to-be-categorized objects and which faces cued the opposite location and that the greater advantage of the valid over the invalid faces in the economic games reflected this awareness. Therefore, in Experiment 4, we also asked participants to complete an extra manipulation check that the gaze-cuing task produced only implicit knowledge of the gaze-cue contingencies (Bayliss et al., 2009). Following debriefing, participants were shown pairs of faces, one valid and one invalid, and asked to indicate those most likely to look toward the location of to-be-categorized objects. Accuracy in this discrimination task might reveal accessible knowledge that the faces differed in their gaze reliability.

## Experiment 4: (True) Dictator Games With Head Movements

### Method

Twenty six healthy adults (13 males and 13 females) completed an amended gaze-cuing task, followed by a series of (true) one-shot dictator games; explicit approachability and trustworthiness ratings; and, finally, a separate discrimination task involving the valid faces and invalid faces as a manipulation check for implicit gaze-cuing. Participants were assessed and debriefed in the same way as Experiments 1, 2, and 3 (Table 1).

#### Amended gaze-cuing task with head-shifts

##### Stimuli

The stimuli were the same as those used in the above experiments; that it to say, photographs of faces approximately 10.6 cm × 10.0 cm, with eyes spanning between 4.0 and 4.5 cm from the left corner of the left eye to the right corner of the right eye. The eyes measured approximately 0.5 cm × 1.0 cm, with pupils/irises of approximately 0.5 cm × 0.5 cm. All faces showed a moderate smile and were initially presented looking straight ahead.

##### Procedure

As before, participants fixated a central cross while covering two response keys with the forefinger and thumb of their dominant hand. Following a pause of 600 ms, the cross was replaced by a face. After another 1,500 ms, the eyes moved to the left or right along with a concurrent translation of the face to the left or right by 0.8 cm (see Figure 1B). Critically, while the gaze direction was linked to whether the face was a “valid” or “invalid” face, the head movement direction was randomly selected. As before, following another 500 ms, a household object appeared on the left or right of the display. Participants were asked to decide, as quickly and accurately as possible, whether the object belonged in the garage (“h” key) or kitchen (spacebar key). Auditory feedback followed: bell to indicate correct; buzzer to indicate incorrect. If no response was made after 2,500 ms, the trial was coded as an error. A blank screen was displayed for a 1,500-ms ITI.

As in Experiments 1, 2, and 3, participants completed 192 trials (two blocks of 96 trials), with eight “valid” faces (with eye gaze shifting toward the side of the to-be-categorized objects) and eight “invalid” faces (with eye gaze shifting away from the side of the to-be-categorized objects) appearing 12 times in a random order, paired with randomly selected targets. For half of the participants, the eight valid faces were taken from Face Group A and the eight invalid faces from Face Group B; for the other half, this assignment was reversed. Participants completed 16 practice trials with a single novel face.

##### Data analysis

Mean correct RTs and error proportions from the gaze-cuing task were analyzed using repeated-measures ANOVAs with the between-subjects factors of participant-gender and face group and the within-subject factor of cue (valid vs. invalid faces). Trials with RTs > 1,500 ms were excluded from analysis. Error proportions were arcsine-transformed; however, the reported values are untransformed percentages.

#### (True) one-shot dictator games (DG)

The one-shot DGs were adapted from the one used in Experiment 3 to capture the essential characteristics of true dictator games (Camerer, 2003a). Participants played a series of one-shot DGs with each of the 16 faces shown as part of the gaze-cuing task above. As before, a valid or invalid face was presented in the center of the computer display above the caption “Here is person *n*,” where *n* equaled 1–16 faces. Two seconds later, participants were prompted to press the spacebar to continue. Following this, participants responded to the question (see Figure S1) “You have £10. How much would you like to give to this person (£0–£10)?” This time, participants were free to allocate as much or as little out of £10 as they wished (whole £s only), but including allocations of 0. Following this, the caption at the bottom of the display was re-presented to state “You have given £*a*, you have kept £10 − *a”* where *a* indicated the amount of money allocated. Three seconds later, a blank screen was presented for an ITI of 3 s. Participants completed four practice games with faces not seen in the gaze-cuing task.

##### Data analysis

Mean allocations in the one-shot DGs were tested with the ANOVA with the between-subjects factor of participant-gender and face group, and the within-subject factor of cue (valid vs. invalid). The numbers of participants making offers to valid faces that were larger, smaller or equal to offers to invalid faces was assessed with a standard χ^2^ test. Mean allocations were tested using one-sample *t* tests against baseline and even-splits of £5 out of £10. Associations between larger allocations to valid compared to invalid faces, gaze-cuing effects and AQ scores were tested with Pearson’s coefficients.

#### Trustworthiness and approachability ratings

Participants were asked to rate the approachability and trustworthiness of the 16 faces presented in the gaze-cuing task in the same way as Experiments 1, 2, and 3.

#### Manipulation check

Finally, participants completed the previously validated manipulation check (Bayliss et al., 2009) to provide further evidence that they were unaware of which faces reliably cued the locations of the to-be-categorized objects (“valid” faces) and which cued the opposite locations (“invalid” faces). Participants were shown eight pairs of faces, each consisting of one valid and one invalid face, and asked to indicate, using the keys “1” and “2” keys, which faces they thought were most likely to have consistently looked toward the side of the display in which object appeared in the gaze-cuing task.

##### Data analysis

Evidence of knowledge of which faces were valid and invalid would be apparent in higher scores, approaching a maximum score of 8. So, scores were transformed as proportions and tested against chance (0.5) with a one-sample *t* test.

### Results

Using the amended gaze-cuing task in which the head (but not the eyes) shifted once to the left or right, participants’ were significantly faster to categorize objects following presentation of valid compared to invalid faces (Figure 2), *F*(1, 22) = 63.33, *p* < .0001, partial η^2^ = .75. Error rates were not much altered (3.65 ± 0.41% vs. 3.33 ± 0.48%; *F* < 1), partial η^2^ < .03.

**Figure 4 fig4:**
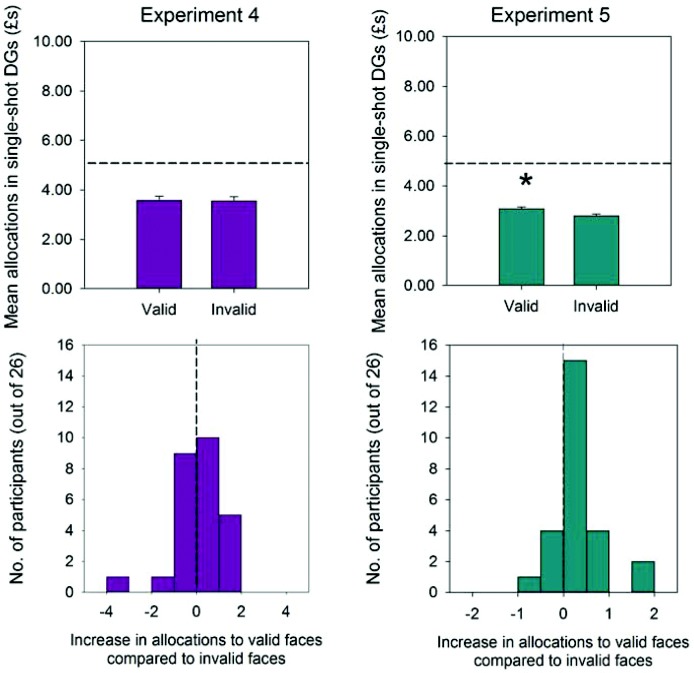
Top row: mean allocations to valid and invalid faces in the one-shot dictator games (DGs) of Experiment 4 (with nonpredictive head translations) and Experiment 5 (with preexposure + nonpredictive head translations). Error bars represent standard errors of the means calculated for within-subject designs (Loftus & Masson, 1994); horizontal dashed lines represent equal offers to valid and invalid faces. Bottom row: frequency of allocation increases (and decreases) to valid compared to invalid faces across the participant samples of Experiments 4 and 5. Vertical dashed lines represent equal allocations to valid and invalid faces. * *p* < .05.

Debriefing indicated that none of the participants in Experiment 4 were aware that some faces always looked in the direction of the to-be-categorized target objects, whilse other faces looked in the opposite direction. In the one-shot DGs, participants’ allocations to both the valid faces and the invalid faces were significantly larger than zero, *t*(26) = 9.64, *p* < .0001, and *t*(26) = 9.15, *p* < .0001, but significantly less than the even-split of £5, *t*(25) = −3.85, *p* < .001, and *t*(25) = −3.75, *p* < .001. However, this time, participants did *not* make significantly more generous allocations to the valid compared to invalid faces (see Figure 4, top row), *F*(1, 22) < 1, partial η^2^ = .00. Although nine participants made larger offers to the valid faces compared to invalid faces, 12 participants made smaller offers and five participants made (overall) equal-sized offers (Figure 4, bottom row; χ^2^ = 2.85, *p* = .24).[Fig-anchor fig4]

The differences in allocations in the true one-shot DGs to the valid faces compared to the invalid faces were not significantly associated with the size of participants gaze-cuing effects (*r* = –.16), or their AQ scores (*r* = –.25). In addition, participants’ offers in the one-shot DGs were not markedly different for male and females (*F* < 1, partial η^2^ = .00) or for the sets of faces assigned as valid or invalid, *F*(1, 22) = 1.45, partial η^2^ < .01.

Participants’ explicit ratings of the approachability and trustworthiness of valid and invalid faces were not significantly different (Table 2; *Fs* < 1, partial η^2^s < .01). Finally, analysis of the manipulation check indicated that participants failed to score significantly above chance (0.51 ± 0.04) when asked to discriminate between valid and invalid faces as those most likely to look toward the location of to-be-categorized objects, *t*(25) = 0.24, *p* = .82.

### Discussion

In Experiment 4, the amended gaze-cuing procedure, in which predictive gaze-shifts were accompanied by small nonpredictive head translations (Bayliss et al., 2005, 2009), produced surprising results. Although this procedure generated robust gaze-cuing effects, the reliability of these gaze cues was not now related to the amount of money allocated to valid faces compared to invalid faces in a series of one-shot (true) DGs. Therefore, while this simple change in the gaze-cuing procedure did not influence the capacity of gaze cues to control spatial attention, it did block the associative processes that link person-identity to gaze reliability. Obviously, encoding person-identity in social encounters where we monitor people‘s gaze depends upon face recognition processes (Johnston & Edmonds, 2009; Young, Newcombe, de Haan, Small, & Hay, 1993). Thus, moving the head (just once) disrupts these processes sufficiently to block learning about whose gaze is, or is, not reliable, at least as manifested in true one-shot DGs.

To test our hypothesis that person identification is disrupted enough to impair the acquisition of knowledge about whose gaze cues are reliable and whose gaze cues are unreliable, we designed our final experiment to facilitate face recognition processes with the intention of restoring the advantage of the valid faces over the invalid faces in subsequent economic games. Experiment 5 was the broadly same as Experiment 4, except that participants were first introduced to the faces that would be used in the gaze-cuing task during a one-back working memory task, as a familiarization procedure. We wished to test the hypothesis that learning associations between face/person-identity and gaze reliability is resistant to perceptual noise (implemented here as head-shifts) where face/person-identity has been preestablished. Thus, in Experiment 5, we predicted restoration of generous allocations of money to valid compared to invalid faces in a series of (true) one-shot DGs. We also asked participants to complete the same valid/invalid face discrimination task as a final manipulation check for implicit gaze-cuing.

## Experiment 5: (True) Dictator Games With Familiar Faces (and Head Movements)

### Method

Twenty six healthy adults (13 males and 13 females) completed a 1-back working memory task with faces, followed by the amended gaze-cuing task involving head translations as in Experiment 4, a series of (true) one-shot DGs, explicit ratings of approachability and trustworthiness and, finally, the discrimination task involving the valid and invalid faces as a manipulation check for implicit gaze-cuing. Participants were assessed and debriefed, as in Experiments 1, 2, 3, and 4 (see Table 1).

#### 1-back working memory task with faces

Participants were asked to monitor the same set of faces to be used in the gaze-cuing task and one-shot DGs, presented with straight gaze in a sequence in the center of the display; and to indicate, using a simple button key-press response, whenever an individual face was presented twice on immediately consecutive trials. The faces were presented in the same dimensions as all of the previous experiments.

On each trial, participants were shown a fixation cross for 500 ms, followed by a face for 1,500 ms. Participants indicated 1-back repetitions using the spacebar. Errors (both false positives and omissions) were indicated by three large red crosses presented for 500 ms prior to the start of the next trial. Two faces were repeated once in each block of 16 trials.

Participants completed eight blocks of trials, seeing each face for a total of nine times (eight times as nonrepetitions and once as a 1-back repeat). Participants were instructed not to monitor for repetitions over the intervals between blocks. The task lasted 6 min. Within each block, faces appeared in a random order. However, two different faces were preselected as repetitions within each block. Participants completed one of four task versions, each defined by the particular faces selected as repetitions within the eight blocks of trials.

### Results

Participants did not record any false-positive errors in the 1-back working memory task. However, there were some failures to recognize face repetitions (omissions; 2.88 ± 0.70%). Then, as in Experiment 4, participants’ were reliably faster to categorize objects following presentation of valid compared to invalid faces (Figure 2), *F*(1, 22) = 19.64, *p* < .0001, partial η^2^ = .47. Errors were not changed (4.52 ± 0.68% vs. 4.22 ± 0.76%; *F* < 1, partial η^2^ < .02).

None of the participants in Experiment 5 were aware that some faces looked in the direction of the to-be-categorized objects whilst others looked in the opposite direction. However, in contrast to Experiment 4, participants once again made significantly more generous allocations to the valid faces compared to invalid faces (Figure 4, top row), *F*(1, 22) < 6.82, *p* < .05, partial η^2^ = .24. Sixteen participants out of 26 allocated more money to the valid relative to invalid faces, while five participants each made smaller or equal-sized offers (Figure 4, bottom row; χ^2^ = 9.31, *p* = .01). Allocations to valid and invalid faces were significantly larger than zero, *t*(25) = 7.21, *p* < .0001, and *t*(25) = 6.54, *p* < .0001, but less than £5 out of £10, *t*(25) = −4.51, *p* < .0001, and *t*(25) = –5.16, *p* < .0001.

Increased allocations, expressed as the difference between those made to valid and invalid faces, were not notably associated with the size of cuing effects (*r* = .01), or AQ scores (*r* = –.02). Participants’ offers were not markedly different for male and females (*F* < 1, η = .00) or for the different sets of faces assigned as valid or invalid (*F* < 1, partial η^2^ < .04).

Participants’ explicit ratings of the approachability and trustworthiness of valid and invalid faces were not significantly different (Table 2; *Fs* < 1, ηs < .01). Finally, analysis of the manipulation check indicated that participants failed to score significantly above chance (0.49 ± 0.04) when asked to discriminate between valid and invalid faces as those most likely to look toward the location of to-be-categorized objects, *t*(25) = 0.40, *p* = .69.

### Discussion

Experiment 4 demonstrated that the simple procedural change of introducing nonpredictive head translations in our amended gaze-cuing task abolished the advantage of the valid faces over invalid faces in a series of (true) one-shot DGs. We hypothesized that these head movements disrupted face recognition processes and impaired the ability of participants to associate particular person/face identities with the reliability of their gaze cues. Experiment 5 provides convincing evidence to support this hypothesis. Familiarizing participants with the faces of the gaze-cuing procedure, using a simple 1-back working memory task, restored their ability to learn which faces reliably looked toward or away from to-be categorized objects. Specifically, participants showed the usual robust facilitation of categorization times following the presentation of already familiar valid faces compared to familiar invalid faces, and then made significantly larger monetary allocations to the valid faces (relative to the invalid faces) in the one-shot DGs.

As in all four other experiments, there were no significant differences in the judged approachability or trustworthiness of the valid faces compared to invalid faces, and participants were not able to report how the valid and invalid faces differed in the amended gaze-cuing task; neither were they able to pick out valid faces as more likely to look in the direction of to-be-categorized objects. Thus, these data support our hypothesis that implicit acquisition of knowledge about gaze reliability is resistant to perceptual noise where person/face identity is preestablished.

## General Discussion

Monitoring and following people’s gaze can be beneficial when joint attention is established over important locations or objects in the environment (Frischen et al., 2007). However, gaze cues can be misleading when they are used, such as by conspecifics competing for valuable resources (Bugnyar & Heinrich, 2006; Bugnyar & Kotrschal, 2004), or by Ronaldhino to misdirect an opponent on the soccer field—encouraging false predictions about future behavior. Here, in Experiments 1, 2, 3, and 5, we showed that following the gaze of reliable social partners can enhance the *behavioral* expressions of social obligations. Gaze-cuing enhances individuals’ propensity to invest money with people whose gaze has been helpful, indicating that gaze-cuing can increase behavioral manifestations of trust. However, gaze-cuing also increases offers to reliable individuals when acting as proposers in the one-shot UGs, suggesting that gaze information heightens obligations to behave fairly even when social partners are encountered just once and would be unlikely to be able to punish attempts to exploit them. Further, gaze-cuing increases allocations of money to individuals with reliable gaze-cue while acting as “dictators” in one-shot DGs, indicating enhanced (painful) altruistic impulses.

Consistent with previous experiments (Bayliss et al., 2009; Bayliss & Tipper, 2006), reliable gaze cues influenced behavior in these economic games without any awareness, on the part of our participants, that some faces reliably cued the spatial locations of the to-be-categorized objects while other faces did not. While completing our gaze-cuing procedure, participants fixated the center of each presented (valid and invalid) face and were quite aware the direction of gaze shifted away from the participants’ face toward the left or the right side of the computer display. However, since their primary task was to rapidly classify the peripheral kitchen and garage objects, the faces were irrelevant and could be ignored. Consistent with multiple experiments (Bayliss et al., 2009, 2010; Bayliss & Tipper, 2006), the irrelevance of the faces to participants’ behavioral goals meant that none of them explicitly recognized the face/person identity-gaze contingencies. Experiments 4 and 5 provided further evidence that our results reflect the operation of implicit social processes: Following completion of the gaze-cuing task and the one-shot DGs, participants were still unable to identify the valid over the invalid faces as those most likely to look in the direction of the to-be-categorized objects. Nonetheless, in Experiment 5, the person-identity contingencies were detected and then utilized by implicit systems to regulate monetary transfers to social partners during the one-shot (true) DGs.

Trait-based models posit that opinions of other people center round character dimensions: warmth/emotion and competence/dominance (Todorov et al., 2008). Here, we found that participants favored the valid faces over invalid faces while playing the economic games of Experiments 1, 2, 3, and 5 even though their explicit ratings of the approachability and trustworthiness of these two sets of faces were not significantly altered. This suggests that the consequences of completing the gaze-cuing procedure upon behavior in economic exchanges are unaccompanied by skewed conscious judgments involving the warmth/emotion character dimensions of the valid relative to the invalid faces encountered in these experiments (Oosterhof & Todorov, 2009; Stirrat & Perrett, 2010).

Our experiments describe an evolving understanding of how gaze-cuing influences implicit social cognitive–affective processes. Previous investigations of gaze-cuing employed forced-choice to demonstrate that valid faces were judged as more trustworthy than invalid faces (Bayliss et al., 2009; Bayliss & Tipper, 2006). Experiment 1 extended those findings by showing that the standard gaze-cuing procedure induced participants to invest more money with the valid faces compared to invalid faces in a series of one-shot Investment/Trust games; indicating enhanced *behavioral* manifestations of trust in persons who have shown reliable gaze cues. However, the investment games of the kind used in Experiment 1 also involve instrumental reasoning about which persons are, or are not, going to be consistently generous trustees in returning investment profits. This left open the possibility that processing gaze information can generate other kinds of social or emotional appraisals including, for example, obligations to be at least fair to people whose patterns of gaze have been useful previously. Accordingly, Experiment 2 used a series of one-shot ultimatum games (Güth et al., 1982) to demonstrate that participants who have completed the gaze-cuing procedure subsequently offered more money to the valid faces compared to the invalid faces. This latter finding suggested that people can develop an enhanced sense of (social) obligation to be fair to social partners who have shown reliable gaze cues in economic transactions.

Taking this line of reasoning one stage further, we noted that proposers in one-shot UGs also need to estimate how large offers need to be in order to induce responders to accept, raising the possibility that larger offers to valid faces over invalid faces in Experiment 2 might still reflect a degree of self-interest. Alternatively, making larger allocations in these games could also be moderated by broader positive or altruistic impulses (van’t Wout et al., 2010). Therefore, Experiment 3 (as well as Experiments 4 and 5) used one-shot DGs (Bolton et al., 1998) to test whether participants would allocate more money to valid faces even when that element of self-interest is removed. Experiments 3 and 5 (but not Experiment 4; see below) provide data to confirm this hypothesis.

Game-theoretic models of economic transactions are effective methods for demonstrating how individuals’ behavior can be nonoptimal in economic exchanges. However, we acknowledge that, in themselves, they do not allow us to isolate specific cognitive processes that would have been influenced selectively by completion of the gaze-cuing procedure. Of course, gaze-cuing probably influences multiple cognitive and social processes operating in each of, and across, the three economic games used here: for example, predicting which faces/persons were more likely to return profits or accept an offer in the investment/trust games and UGs of Experiments 1 and 2; impulses to behave fairly in the UGs of Experiment 2 or make altruistic offers in the DGs of Experiments 3 and 5. However, notwithstanding these possibilities, the most parsimonious interpretation of our findings is that acquisition of implicit knowledge of which individuals show reliable gaze cues prompts the expression of approach-based behavior up to, and including, painful altruism in one-shot DGs. Our data also complement other findings that generous offers in ultimatum games can be prompted by the trustworthiness of responders judged on the basis of facial characteristics and coded by anterior insula cortical signals (Kim, Choi, & Jang, 2012).

Although the effects of gaze-cuing in economic games appear robust, generalizing across different procedures in five experiments, we have also identified an important boundary condition. Specifically, when the faces provided both predictive cues (as movements of the eyes to indicate gaze shifts) and nonpredictive cues (as small translations of the head), typical cuing effects were obtained but no learning of the relationship between person identity and gaze reliability was detected. Thus, subtle interference of face identification processes produced by a small degree of perceptual noise, such as a single head shift to the left or right, is quite disruptive. Other evidence indicates that the link between face-identity and gaze-cuing can be weak (Frischen & Tipper, 2004); in some cases, gaze-cuing effects being only enhanced with famous valid and famous invalid faces (Frischen & Tipper, 2006). Similarly, the emotional expression of the faces moderates the acquisition of implicit knowledge of reliable gaze cues so that happy but not disgusted (Bayliss et al., 2007), angry, or neutral (Bayliss et al., 2009) faces elicit this form of social learning.

The finding that gaze-cuing can be enhanced, or persist in memory, with famous faces (Frischen & Tipper, 2006), suggested that it might be possible to restore the prosocial effects of gaze-cuing in economic games even in the presence of head-shifts by preexposing the faces in a simple 1-back working memory task. Experiment 5 decisively confirms this prediction. These data indicate that the output of neural networks that encode (facial) identity is made available to overlapping systems that control gaze-evoked shifts of attention in order to integrate the reliability of gaze shifts over multiple encounters. Our demonstration of this associative interplay between identity, gaze-evoked shifts of attention and reliability is all the more remarkable given that both face identity and gaze orientation were irrelevant to the kitchen/garage object classification task. Participants were told to ignore the faces and, as shown previously (Bayliss et al., 2009; Bayliss & Tipper, 2006), were consistently unaware of the identity-gaze reliability connection. Thus, it is unsurprising that implicit associative processes can be disrupted by perceptual noise, at least in some situations.

Finally, we note two outstanding empirical issues. First, the present data do not indicate whether gaze-cuing heightens altruistic impulses toward individuals with reliable gaze cues—operationalized here as “valid” faces—or diminishes such impulses toward individuals with unreliable or misleading gaze cues—operationalized as the “invalid” faces. Future experiments could explore these possibilities by comparing behavior in economic games involving valid and invalid faces with behavior involving “neutral” faces that are as likely to look toward the to-be-categorized object as they are to look away from them.

Second, we do not yet know whether the effects of implicit social processes upon economic games are specific to clearly social cues such as eye gaze, or whether other cues—consistent but devoid of overt social content—would generate similar effects. For example, one manipulation might pair some faces and a hat with a peak that consistently point toward or away from the location of to-be–categorized objects. Possibly, as in the case of symbolic cues such as arrows (Tipples, 2002), such cues would facilitate the categorization of objects as we find with standard gaze cuing. However, in other work, we have shown that, while such cues can reorient viewers’ spatial attention, they do not engage the same social cognitive processes as directed gaze (Bayliss et al., 2006). Moreover, gaze-cuing effects appear to be sensitive to agency, suggesting a role for theory of mind processes in gaze-cuing itself (Teufel, Alexis, Clayton, & Davis, 2010). Hence, the extant evidence strongly suggests that nonsocial cues associated with individual persons or faces would not influence economic exchanges in the way observed here with gaze cues.

In summary, our experiments investigated whether implicit learning about gaze influences behavior in economic games. The results show that people will tend to make enhanced investments in a series of one-shot investment/trust games with social partners’ whose gaze cues have been reliable previously; they will also make enhanced offers in one-shot UGs and more generous allocations in one-shot DGs. These behaviors reveal altruistic impulses toward social partners with helpful gaze cues. However, the tendency to favor partners with reliable gaze cues over those with unreliable gaze cues can be abolished by perceptual noise that disrupts person-recognition processes, indicating the effects of gaze-cuing in economic exchanges depends upon the interplay of systems that encode identity and control gaze-evoked shifts of visual attention, integrating the value of gaze-shifts over multiple encounters.

## Supplementary Material

10.1037/a0033677.supp

## Figures and Tables

**Table 1 tbl1:** Demographic and Psychometric Characteristics of Participant Samples in Experiments 1–5

Experiment	*N*	Male:Female	Age (years)		Mill Hill Vocabulary		BDI		Autism Spectrum Quotient	
			*M*	*SE*	*M*	*SE*	*M*	*SE*	*M*	*SE*
1	20	10:10	29.30	2.45	40.75	1.46	3.40	0.68	15.65	1.59
2	20	10:10	22.15	0.82	32.00	2.09	4.90	0.63	16.50	1.26
3	20	10:10	21.60	0.66	32.20	1.87	5.55	1.61	18.45	1.48
4	26	13:13	22.62	0.92	33.00	1.68	5.35	0.81	15.15	0.83
5	26	13:13	23.38	0.66	36.62	1.74	5.65	1.13	15.85	1.26
*Note.* Mill Hill Vocabulary = Mill Hill Vocabulary Scale (Raven, Court, & Raven, 1998); BDI = Beck Depression Inventory (Beck, Ward, Medelson, Mock, & Erbaugh, 1961); Autism Spectrum Quotient = score on the Autism Questionnaire (Baron-Cohen, Wheelwright, Skinner, Martin, & Clubley, 2001).										

**Table 2 tbl2:** Explicit Ratings of Approachability and Trustworthiness of the Valid Faces and Invalid Faces Presented in the Gaze-Cuing Tasks of Experiments 1–5

Experiment	Approachability		Trustworthiness	
	Valid faces	Invalid faces	Valid faces	Invalid faces
1	5.10 ± 0.20	5.03 ± 0.19	4.36 ± 0.20	4.51 ± 0.16
2	4.45 ± 0.22	4.58 ± 0.23	4.46 ± 0.20	4.39 ± 0.21
3	4.72 ± 0.23	4.56 ± 0.27	4.05 ± 0.25	3.77 ± 0.25
4	4.72 ± 0.19	4.63 ± 0.24	4.17 ± 0.24	4.38 ± 0.43
5	4.47 ± 0.26	4.50 ± 0.23	4.14 ± 0.21	4.10 ± 0.19
*Note.* Participants provided ratings using a Likert-type scale of 0 to 7.
