# Supplementary material for: I Want to Help You, But I Am Not Sure Why: Gaze-Cuing Induces Altruistic Giving
Source: J Exp Psychol Gen. 2013 Aug 12;143(2):763–77. doi: 10.1037/a0033677 (PMC3970851; doi:10.1037/a0033677)

**­­**

**Supplemental Figure S1.** Invitation displays for investments in the Investment/Trust games (Experiment 1); for offers and allocations in the one-shot Ultimatum Games (UGs) and adapted one-shot Dictator Games (DGs) of Experiments 2 and 3; and for the (true) one-shot Dictator Games of Experiments 4 and 5.


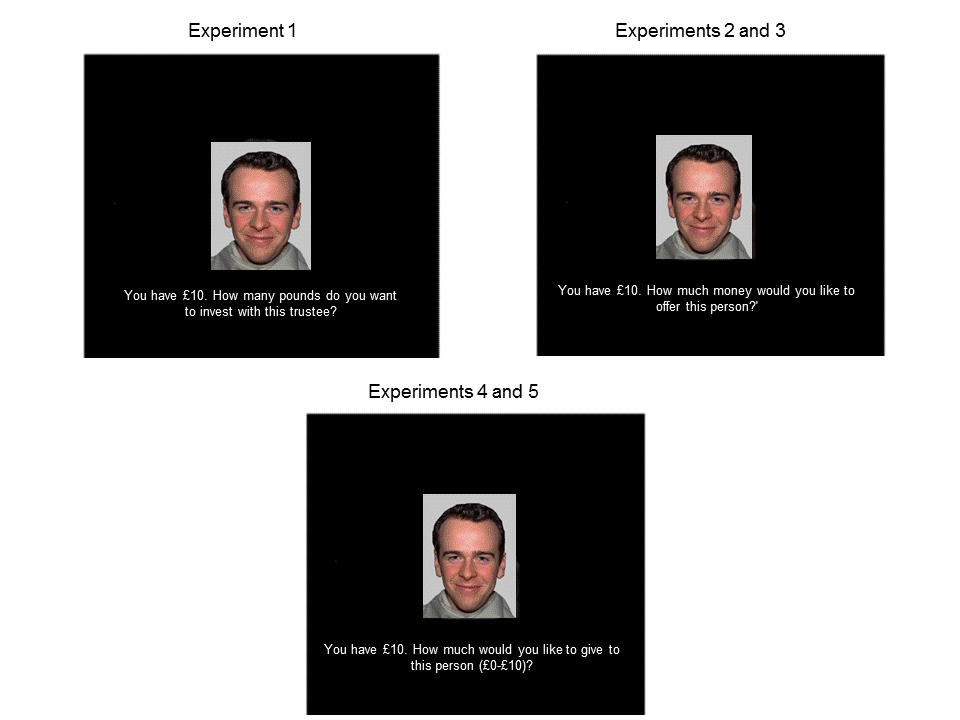

Supplement: Supplementary file 1 [file Gaze_exchange_JEP-G_supps_v1_31_01_13.docx]
